# Parental perspectives on retention and secondary use of neonatal dried bloodspots: a Dutch mixed methods study

**DOI:** 10.1186/s12887-019-1590-8

**Published:** 2019-07-09

**Authors:** Marleen E. Jansen, Lion J. M. van den Bosch, Marjolein J. Hendriks, Mariska M. J. Scheffer, Marie-Louise Heijnen, Conor M. W. Douglas, Carla G. van El

**Affiliations:** 1 Amsterdam UMC, Vrije Universiteit Amsterdam, Department of Clinical Genetics and Amsterdam Public Health research institute, Section Community Genetics, de Boelelaan 1117, Amsterdam, the Netherlands; 2 National Institute for Public Health and the Environment (RIVM), Centre for Health Protection, Postbus 1, Bilthoven, 3720 BA the Netherlands; 3 National Institute for Public Health and the Environment (RIVM), Centre for Population Screening, Postbus 1, Bilthoven, 3720 BA the Netherlands; 4 Department of Science and Technology Studies, Faculty of Sciences, York University, 307 Bethune College, 4700 Keele St, Toronto, ON M3J 1P3 Canada

**Keywords:** Neonatal dried bloodspots, Retention, Secondary use, Parental perspectives, Policy-making

## Abstract

**Background:**

Neonatal bloodspot screening (NBS) identifies conditions to offer early intervention and minimize irreversible damage. NBS policies guide a comprehensive system including processes for storage of neonatal dried blood spots (NDBS). NDBS retention and secondary use policies have been subject of public debates internationally, suggesting that the public’s perceptions of NDBS policy are not always on par with existing policies. The current study aims to provide insight in relevant factors for new parents in the Netherlands regarding retention and secondary use of NDBS. These factors can be taken into account when developing or updating NDBS policies.

**Methods:**

A mixed methods design was used combining an online survey (*n* = 753), focus groups (6 groups, 37 participants), and individual in-depth interviews (*n* = 7). The discussed topics included: parental information, obtaining informed consent, support for retention, and support for secondary use. The study population consisted of Dutch-speaking new parents: pregnant women (≥20 weeks) and/or their partner, and parents of at least one child (≤5 years).

**Results:**

New parents expressed needs for easily accessible information, adequate communication on the retention and (potential) use of NDBS, clearly described safeguards for privacy, a more active consent process, regulation for the actors conducting NDBS research, and parental involvement in decisions on secondary use. Overall, participants were positive about prolonged retention and different types of secondary use if those needs were met.

**Conclusions:**

While parental involvement is a challenge, our study is an example of gauging parent’s perspectives on NDBS policy and contributes to including these perspectives in the current policy discussion on longer retention. Prolonged retention could be a feasible option in the Netherlands if several prerequisites are met. Therefore, implementation studies involving parents are needed.

**Electronic supplementary material:**

The online version of this article (10.1186/s12887-019-1590-8) contains supplementary material, which is available to authorized users.

## Background

Most neonatal bloodspot screening (NBS) programs identify a selection of rare, serious, congenital conditions for which timely detection and treatment early in life prevents or minimizes irreversible damage [1]. NBS has developed into a complex system with the potential of detecting over 50 conditions. To ensure an acceptable, relevant, and responsible NBS program for the target population, policy decisions are generally based on all aspects included in the Wilson and Jungner screening principles [2–4]. While the Wilson and Jungner principles do not include guidance for storage of neonatal dried blood spots (NDBS), most countries have such processes in place, for example as part of quality control [5]. NDBS retention and secondary use policies have been subject of public debates internationally [6–9], suggesting that the public’s perceptions of NDBS policy are not always on par with current policies.

In the Dutch NBS program (Table 1) NDBS are stored for one year to allow for quality control, such as verification of test results [11]. After one year, most Dutch NDBS are stored for an additional four years. Storage of NDBS beyond the time needed for quality control is called retention. Retention of NDBS serves secondary goals, such as biomedical research into current and future NBS conditions, and paediatricians can request NDBS for diagnostic purposes for individual children after consent of the parents [6, 12]. However, since the studied conditions are rare, retention beyond five years is currently under consideration to facilitate biomedical research [13].

**Table 1:** Neonatal screening program in the Netherlands summarized

Screening process	Conditions in the current program	Conditions included in the expansion
All new Dutch parents are offered NBS for their child. Annually, over 99% of approximately 172,000 Dutch neonates undergo NBS [10]. Similar to other NBS programs, a few drops of blood are obtained from the heel and collected on a filter paper card between 72 and 168 h postpartum. The cards with the dried bloodspots are sent to one of five Dutch regional screening laboratories and analyzed for biochemical markers. After analyses, the NDBS cards are pseudonymized through an encrypted barcode and stored centrally at the national reference laboratory. In 2017, the national NBS program has been expanded from 17 to 19 conditions. It is expected to be expanded with 12 additional conditions within five years. The NBS program is directed by the Centre for Population Screening at the RIVM.	1. Alpha-thalassemia (HbH-disease) 2. Beta thalassemia major (TM) 3. Biotinidase deficiency (BIO) 4. Congenital adrenal hyperplasia (CAH) 5. Congenital hypothyroidism (CH) 6. Cystic fibrosis (CF) 7. Galactosemia (GAL) 8. Glutaric acidemia type I (GA-1) 9. HMG-CoA-lyase deficiency (HMG) 10. Isovaleric acidemia (IVA) 11. Long-chain hydroxyacyl-CoA dehydrogenase deficiency (LCHADD) 12. Multiple CoA Carboxylase deficiency (MCD) 13. Maple syrup urine disease (MSUD) 14. Medium-chain acyl CoA dehydrogenase deficiency (MCADD) 15. 3-Methylcrotonyl-CoA carboxylase deficiency (3-MCC) 16. Phenylketonuria (PKU) 17. Sickle cell disease (SCD) 18. Type 1 tyrosinemia (TYR-1) 19. Very long-chain acylCoA dehydrogenase deficiency (VLCADD)	20. Carnitine-acylcarnitine translocase deficiency (CACT) 21. Carnitine palmitoyltransferase deficiency type 1 (CPT1) 22. Carnitine palmitoyltransferase deficiency type 2 (CPT2) 23. Galactokinase deficiency (GALK) 24. Guanidinoacetate methyltransferase deficiency (GAMT) 25. Methyl-acetoacetyl-CoA thiolase deficiency, ketothiolase deficiency (BKT) 26. Methylmalonic acidemia (MMA) 27. Mucopolysaccharidosis type 1 (MPS I) 28. Organic cation transporter 2 (OCTN 2) 29. Propionic acidemia (PA) 30. Severe combined immune deficiency (SCID) 31. X-linked adrenoleukodystrophy (ALD)

*NBS* neonatal bloodspot screening, *NDBS* neonatal dried bloodspot, *RIVM* Dutch National Institute for Public Health and the Environment

Retention only takes place in the Netherlands if parents did not opt-out from retention and secondary use of their child(ren)‘s NDBS [6]. The opt-out process is done at the time of NDBS collection (72–168 h postpartum, Table 1). The screener fills out the information on the blood spot card, including the answer to the tick box “Parent objects to storage of blood spot for anonymous scientific research.” Additionally the parent has to sign if he or she objects to retention and secondary use, and does not sign if he or she accepts. Secondary use in biomedical research only takes place after approval of the Dutch Research Committee on Neonatal Screening at the National Institute for Public Health and the Environment (RIVM). Secondary use requests are considered if the goal can solely be achieved using NDBS and if the request aims to contribute to public health through prevention of disease or improvement of treatment [7, 14, 15].

Internationally, NDBS policies were often implemented without public debate on what would be acceptable, relevant, and responsible policy [13]. Public commotion about retention of NDBS emerged after the public became aware of various forms of secondary uses [6, 16]. In the Netherlands in 2000, media coverage made clear that also the general Dutch public were not aware of the indefinite retention of the cards [6, 16, 17]. The policy of indefinite storage was deemed inappropriate, and the current maximum storage period of five years was implemented, but without public debate on appropriate NDBS policy [13]. However, public involvement is increasingly considered a core aspect of health policy-making [18–20]. Since new parents decide on NBS for their child(ren) we argue that their perspectives should be taken into account to construct NDBS policy [11].

Factors that contribute to parental support towards retention and secondary use of NDBS include adequate parental information, altruism, and trust in the responsible authority [21–24]. Previous research among new Dutch mothers (*n* = 1272) showed about 70% of the respondents had a positive perception of prolonged retention of NDBS [16]. However, it remains unclear what factors influence the perspectives of new Dutch parents on NDBS retention and secondary use policy, to what extent, and how they are associated. Therefore, this study aims to provide insight in current new parents’ perspectives on NDBS policy. These factors can be taken into account when developing or updating NDBS policies.

## Methods

### Study design

A mixed methods design was used combining an online survey developed by Van Teeffelen et al. (2016), focus groups (FG) and individual in-depth interviews [16, 25]. The FG were semi-structured using two non-directional scenarios [26]. (Additional file 1) The topics in the survey were used to develop the scenarios for the FG and the topic list for the individual interviews to ensure that all research approaches included the same high-level themes: parental information, obtaining informed consent, support for retention, and support for secondary use.(Additional file 2) Since we used different research approaches, not all topics were covered in the same way. For example, in the survey and individual interviews we asked if respondents and interviewees objected to retention and secondary use. Due to the group dynamics this question was considered a sensitive topic and therefore unsuitable for the FG. The Medical Ethical Committee of VU University Medical Centre Amsterdam decided that this study is not subject to the Medical Research Involving Human Subjects Act (WMO) (reference A2011.040 and A2017.141). All FG-participants and interviewees were informed and gave informed consent prior to participation.

### Study population

The study population consisted of Dutch-speaking new parents: pregnant women (≥20 weeks) and/or their partner, and parents of at least one child (≤5 years). Respondents and participants for the survey and FG were recruited through social media (February–May 2017). Survey respondents that indicated interest for additional research received an e-mail (*n* = 273) to participate in FG or an interview. FG-participants were additionally approached via midwifery practices, yoga schools, and day-care centres in four Dutch regions. To reach data saturation six FG and seven interviews were organized.

### Data collection

A 15-min survey was accessible online (March–April 2017). Respondents could sign up for a raffle to receive one of ten vouchers (€25.00). FG (April–May 2017) lasted approximately 2 h. LvdB, CvE, and MH moderated the FGs, and an assistant was present to observe and take notes. Each FG-participant received a voucher (€20.00), a small gift for their (unborn) child, and travel reimbursement. Each phone interview, conducted by MS, took between 20 and 30 min and a summary of the interview was sent to each interviewee for revision.

### Data analysis

From the survey, descriptive statistics were summarized and chi-squared tests were used. Knowledge on current NDBS policy and trust in the RIVM were tested against perceptions of respondents (level of agreement with several retention periods, different types of secondary use, and different actors conducting the research), stratified for socio-economic status. A *p*-value of 0.05 was considered statistically significant (SPSS 22.0) [27].

Interviews were audio-recorded, transcribed verbatim, and coded (ATLAS.ti 8.0). Thematic content analysis was applied to FG, and content analysis using codes based on the survey and FG was applied to the interviews. Two transcripts were selected from both FG and interviews and coded independently (MH and MS) to check for consistency. The coding list (Additional file 3) was updated based on discussions with the research team until consensus was reached [28].

## Results

The survey received 753 eligible responses, FG were conducted with 35 newly recruited participants and two survey respondents, and the interviews were held with seven survey respondents (Table 2).

**Table 2:** Characteristics of the participants^a^

	Survey	In-depth interviews	Focus groups (*n* = 6)
Participants, n (range)	753	7	37 (3–12)
Sex, n (%)			
*Female*	732 (97.2)	6 (85.7)	34 (91.9)
*Male*	18 (2.4)	1 (14.3)	3 (8.1)
*Not answered*	3 (0.4)	0 (0)	0 (0)
Age, n (%)			
*< 30*	219 (29.1)	2 (28.6)	6 (16.2)
*30–35*	326 (43.3)	5 (71.4)	21 (56.8)
*> 35*	205 (27.2)	0 (0.0)	10 (27.0)
*Not answered*	3 (0.4)	0 (0)	0 (0)
Pregnant, n (%)	159 (21.1)	2 (28.6)	9 (24.3)
Number of children			
*0*	46 (6.1)	3 (42.6)	9 (24.3)
*1*	375 (49.8)	1 (14.4)	15 (40.5)
*2*	239 (31.7)	2 (28.6)	11 (29.7)
*> 2*	93 (12.4)	1 (14.4)	2 (5.4)
Nationality^b^			
*Dutch*	684 (90.8)	7 (100.0)	34 (91.9)
*Other*	68 (9.0)	0 (0.0)	3 (8.1)
*Not answered*	1 (0.1)	0 (0)	0 (0)
Education level^c^			
*Low*	9 (1.2)	0	0 (0)
*Medium*	303 (40.2)	0	9 (24.3)
*High*	439 (58.3)	7 (100.0)	28 (75.7)
*Other*	2 (0.3)	0 (0)	0 (0)

^a^FG were conducted with 35 newly recruited participants and two survey respondents, and the interviews were held with seven survey respondents

^b^Does not add up to 100% for the survey respondents due to rounding

^c^High: higher vocational training, university; Medium: higher level of secondary school, intermediate vocational training; Low: elementary school, lower level of secondary school, lower vocational training

### Parental information on retention and secondary use

Individual interviewees, FG-participants and survey respondents indicated that they would like to receive information on retention and secondary use (see below and Table 3). During pregnancy and shortly after childbirth, new parents receive a leaflet about NBS, including general information on retention and secondary use [29]. However, many FG-participants did not remember receiving the leaflet. When knowledge in the survey was analysed, it was found approximately a quarter of respondents had full knowledge (Table 4).

**Table 3:** Survey outcomes on parental information. (*n* = 747) Statements were scored on a five point scale (strongly disagree – strongly agree)

	I would you like to receive information on retention and secondary use…		
	when my child’s NDBS is potentially used in secondary research, so I can give permission. (Strongly) agree, *n (%*)	about the results of the secondary use research project. (Strongly) agree, *n (%*)	when the storage length changes. (Strongly) agree, *n (%*)
I trust the RIVM-committee to take the right decision on secondary use of NDBS.			
(*n* = 572,76.6%, (strongly) agree)	347 (60.7)	426 (74.2)	400 (69.8)
(*n* = 69, 9.2%, (strongly) disagree)	55 (79.7)	54 (78.3)	56 (81.2)
The transparency of the management of the NDBS is insufficient.			
(*n* = 343,45.9%, (strongly) agree)	254 (74.1)	278 (81.0)	284 (82.8)
(*n* = 125, 16.7%, (strongly) disagree)	61 (48.8)	84 (66.7)	73 (57.9)

**Table 4:** Survey outcomes on knowledge^a^. (*n* = 753)

Were you aware that…		Yes, n (%)	No, n (%)
1	…the blood from the heel prick is collected on a paper card, the so-called heel prick card?	719 (95.5)	34 (4.5)
2	…all cards are destroyed after a maximum of 5 years?	208 (27.6)	545 (72.4)
3	…parents can object to the storage of their child’s card for research?	365 (48.5)	388 (51.5)

^a^Full knowledge was defined as “yes” on all three items, medium knowledge as “yes” on item 1 and item 2 or 3, little knowledge as “yes” on item 1, no knowledge as “no” on all three items

#### Content of parental information

Survey respondents were asked if they would like to receive information for three situations: 1) when their child’s NDBS is potentially used in secondary research, 2) about the results of the secondary use research project and 3) when the storage length changes. Approximately half of them wanted to receive information for all situations. Both FG-participants and interviewees would like to receive information on retention and secondary use, but in many FG it was also mentioned that the information currently provided is not adequate.

*“I would like to receive information on both the use of my child’s NDBS and the study results. I think providing information is one of the pillars of scientific research, therefore, information about the use of NDBS and the study results should be transparent at all times.” - (M, Interview 5)*.

*“There should be more information. If people have a certain fear of not doing something, information can achieve a lot. Indeed, by giving examples of 'this and this' could be done with [NDBS].” - (F, FG 2)*

Perceived transparency of the NDBS management (χ^2^ = 15.82, *p* = 0.000) and trust in the current decision-making process (χ^2^ = 38.56, *p* = 0.000) were associated with whether survey respondents wanted to receive information on all three situations versus information on one or two situation(s) or no information. People who had lower trust in the current decision-making process and perceived low transparency of the NDBS management wanted to receive more information (Table 3).

Besides general information on NDBS in the NBS leaflet, FG-participants suggested access to more detailed information about research purposes for secondary use to increase support.

*“But you see it more often, just like vaccinations, parents are willing, but don’t feel adequately informed. […] For me that is the same here, I want to [give consent for retention and secondary use], but I am not informed properly.” (F, FG 1)*

Many new parents in FG and interviews believed it would be sufficient if access to information on secondary use is facilitated for example on the RIVM-website. Informing parents when their child’s NDBS is used, is mentioned several times, but would entail a link to personal information, which interviewees considered worrisome with regard to anonymity of the samples. A majority of the survey respondents also indicated they would like to have access to information on results of secondary use on the RIVM-website (*n* = 578, 76.8%).

#### Timing of information

The NBS leaflet that includes information on retention and secondary use of NDBS is currently handed out twice: at approximately 36 weeks of gestation at the midwifery practice or hospital, and when the newborn child is registered at the municipality [29]. The timing was not included in the survey nor discussed in the interviews. According to FG-participants, the timing of the information could be shifted to earlier in the pregnancy to give new parents more time to consider retention and secondary use.

*“I think parents have enough on their mind just after the child is born. Let me just say it quite honestly: you have so much to deal with and then you have to think about this and then about that. And shouldn’t [information] be provided earlier? Not everyone makes it to 36 weeks.” (F, FG 1)*

In contrast, other FG-participants argued earlier information would increase the likelihood of forgetting, and supported the current information timing. Other FG-participants indicated information should be provided later, during regular check-ups at a child healthcare centre. In that case consent for retention and secondary use should also be obtained at a later time point.

### Obtaining consent

Consent for retention and secondary use is currently obtained with an opt-out approach at the time of the bloodspot collection.

#### Informed consent approach

Some FG-participants preferred the current opt-out approach, where parents only sign if they object. Many FG-participants preferred an opt-in approach, where parents need to sign regardless if they consent to retention and secondary use or not. While in the survey 71.6% (*n* = 539) of the respondents supported the current opt-out procedure, 40.5% (*n* = 303, 4 missings) preferred an opt-in approach. According to some individual interviewees and FG-participants, not everyone takes an informed decision or makes an active choice with an opt-out approach. FG-participants also felt that at the first few days postpartum it is difficult to make an informed decision.

*“I think when you sign [in both cases], then you see which box [yes or no for four years retention and anonymous scientific research] is ticked, that would be better for me. Then I see: well, ‘no or yes has been selected, that is the answer I gave, then it is alright’.” (F, FG 4)*

The survey showed that half of respondents know that they could object to retention and secondary use (Table 4). Another suggestion by several FG-participants was to add options for types of secondary use on the card, which could each be consented to or not. Arguments of FG-participants supportive of an opt-out approach included that while the consent procedure seems more passive, new parents are still not obliged to consent. Some FG-participants considered an opt-out approach easier than opt-in for the screener. Furthermore, these FG-participants expected a higher participation rate for secondary use with this opt-out approach compared to an opt-in approach.

*“I think there will be more NDBS available for research when people don’t have to sign [for permission].” (F, FG 2)*

Some FG-participants supported opt-out when they would have access to detailed information about secondary use of NDBS.

### Support for retention

Results from the survey showed 66.1% of the respondents did not object or were not planning to object to current NDBS retention and secondary use because they think scientific research is good (Table 5, *n* = 497). Not objecting is not associated with socio-economic status (*p* = 0.507) or level of education (*p* = 0.170), but is associated with knowledge on the current NDBS policy (*p* = 0.023) (Table 5). FG-participants were not asked if they objected to retention and secondary use, none of the individual interviewees objected to retention and secondary use.

**Table 5:** Survey outcomes on support. (*n* = 753)

	Did you or are you planning to object to retention and secondary use?				*p*-value
	No, I think scientific research is good.^c^ (*n* = 497, 66.1%)				
Knowledge on current NDBS policy *(no, little, medium, full)*	24 (4.8)	129 (26.0)	199 (40.0)	145 (29.2)	0.023
SES *(low, medium, high)*^a^	69 (14.2)	315 (64.7)	103 (21.1)		0.507
Level of education *(low, medium, high)*^b^	5 (1.0)	191 (38.6)	299 (60.4)		0.170
	Bad	Somewhat bad	Not bad, not good	Somewhat good	Good
What do you think of use of the heel prick card of your child for research that potentially improves public health but does not benefit you child directly? *n (%)*	16 (2.1)	11 (1.5)	48 (6.4)	112 (14.9)	566 (75.2)
As far as I am concerned, the stored heel prick cards can be used to…	Disagree	Somewhat disagree	Neutral	Somewhat agree	Agree
…determine how often certain diseases occur here	39 (5.2)	12 (1.6)	36 (4.8)	98 (13.0)	568 (75.4)
…figure out how certain diseases develop	25 (3.3)	12 (1.6)	21 (2.8)	73 (9.7)	622 (82.6)
…allow universities to develop medical tests	45 (6.0)	11 (1.5)	48 (6.4)	96 (12.7)	553 (73.4)
…allow universities and companies together to develop medical tests	85 (11.3)	52 (6.9)	94 (12.5)	157 (20.8)	365 (48.5)
…allow companies to develop medical tests	183 (24.3)	94 (12.5)	112 (14.9)	112 (14.9)	252 (33.6)
…help trace suspects of serious crimes	243 (32.3)	48 (6.4)	92 (12.2)	78 (10.4)	292 (38.8)
…identify victims of fire, natural disaster or kidnapping	73 (9.7)	24 (3.2)	54 (7.2)	102 (13.5)	500 (66.4)
…connect to my personal medical dossier for research into diseases in my family	86 (11.4)	24 (3.2)	72 (9.6)	93 (12.4)	478 (63.5)
…connect to my personal medical dossier for research into diseases in the Dutch population	151 (20.1)	54 (7.2)	85 (11.3)	112 (14.9)	351 (46.6)

^a^10 missing, *n* = 487

^b^2 missing *n* = 495

^c^The answer chosen by most survey respondents

#### Retention period

Most survey respondents (*n* = 493, 65.5%, Table 6), FG-participants, and interviewees supported longer retention compared to current retention. Many FG-participants considered 16 years ideal, based on administrative, ethical, and legal aspects, such as the child’s autonomy.

*“I don’t think you should go over 16 years. Unless the child would give consent again, but then you will be in such an administrative hassle. Yes, it will be such an administrative burden that I don’t think that is practically feasible.” (F, FG 3)*

**Table 6:** Survey outcomes on retention period. (*n* = 753)

In the current system of centralized storage and anonymous use, the NDBS can maximally be stored … n (%)	1 year	5 years	18 years	indefinitely	No opinion	Other
	59 (7.8)	153 (20.3)	115 (15.3)	378 (50.2)	32 (4.2)	16 (2.1)

A frequently asked question by FG-participants and interviewees was whether longer retention would add value, because that would determine whether they would support longer retention. Also interviewees mentioned uncertainty about the added value, but stressed that the child should be able to decide at some point in time.

*“In my opinion, the storage period should be extended as long as they need it. I have no idea how long the material remains useful.” - (F, Interview 2)*

On the other hand, worries on prolonged retention were shared in FG, such as unforeseen developments in politics, legislation and technology.

*“The legislation can change and technology goes so much faster. And if you store something for 16 years, I have no idea what the world looks in 16 years and what they will be able to do.” (F, FG 4)*

#### Management of retention

NDBS retention is currently managed and facilitated by the RIVM, which was supported by FG-participants, interviewees, and half of the survey respondents (50.3%, *n* = 410, *not shown in* Table 5). In FG and interviews privacy concerns were raised. Most participants did not consider the current storage as fully anonymous, because the cards can still be traced through a barcode. FG-participants voiced worries about cybercrime related to digital storage of personal data.

*“Not that something would happen, but for me personally, [cybercrime] would be just a very scary idea. Suppose that one of [my children] has a disease,[and then] that’s just out in the open, because you never know when you get it back, sort of say. Once it’s open and exposed. Yes, it may be that it’s really a fear and that I see ghosts everywhere or whatever, but that is why I find this [longer retention and secondary use] so interesting.” (F, FG 6)*

Also in the interviews, some interviewees perceived risks of leaking of sensitive information from NDBS for example to insurance companies. A suggested solution from FG-participants for anonymity and privacy issues was to add an extra blood spot, which would be stored separately for anonymous research.

### Support for secondary use

Both secondary use for biomedical research to improve NBS and general public health research were highly supported by FG-participants, interviewees, and respondents. The latter scored with a large majority “somewhat good” or “good” on ‘What do you think of use of the heel prick card of your child for research that potentially improves public health but does not benefit you child directly?’ (*n* = 678, 90.1%, Table 5). Arguments of both FG-participants and interviewees to support biomedical research included health benefits and increasing scientific knowledge.

*“The moment you have (collected) the blood, do something with it. If the card is discarded, yes I find it almost a waste to throw it away. I think as long as the scientific and medical world might learn from it, and can achieve something with it, it is always valuable to use it” - (F, Interview 1)*

Alternative purposes for secondary use, such as victim identification, were generally supported in all research arms (Table 5), but concerns for privacy were mentioned when the secondary use was beyond anonymous research. Some FG-participants did not have any concerns, others showed concerns about DNA-biobanking and breaching anonymity to enable victim identification and suggested other available options for identification. Furthermore, FG-participants and interviewees considered it would be only a small step away from identification of criminals, which was least favoured (Table 5).

*“I would be very unhappy it if someone suddenly shows up at my door and says, we linked the DNA of your son’s heel prick card (to a certain crime). […] What kind of influence has this on our privacy? How will people judge us when this happens? However, it depends on why they are looking for someone.” - (F, Interview 2)*

Secondary use can be conducted in research projects by different organizations. Survey respondents and FG-participants indicated that they did not fully support secondary use by companies (Table 5). According to FG-participants, companies may have ‘wrong intentions’, such as using the samples solely for commercial purposes, which was also mentioned by individual interviewees.

*“I think that it [research with NDBS] has to have healthcare purposes. It is fine if it [NDBS] goes to anywhere, but it should not be the case that a company profits commercially and it has no health benefit” (F, FG 3)*

On the other hand, secondary use of NDBS by universities or governmental organizations that serve public health was highly trusted by interviewees, FG-participants and survey respondents (Table 5). Worries about NDBS falling into the wrong hands, were also shared. Both FG-participants and interviewees were concerned about costly insurance or obtaining insurance at all.

*“Insurance companies that, at a certain moment, when you are twenty-five, tell you that you cannot buy a house. Like, you get nothing because we already know about the heel prick card, you do not know it yet, but we already know that you have a degenerative disease or something.” - (F, Interview 1)*

Current decision-making on secondary use includes a multidisciplinary committee including a medical, ethical and patient perspective. Table 3 shows that 76.6% of the survey respondents trust the decision-making process in place to result in suitable research projects. Results showed different degrees of support for parental involvement in the decision process on secondary use. Survey data showed parents or parent representatives were preferred by 285 (37.8%) survey respondents, and an independent committee including parents, patients, and experts by 232 (30.8%), while 187 (24.8%) preferred the current committee. Most FG-participants and interviewees supported some involvement of parents in the decision-making process on secondary use.

*“It seems like a good idea to involve parents [in the committee]*… *Not that it (the committee) should consist of parents only*… *Parents must also be included, like you involve them in your research right now, and they must be well represented*…*In a careful manner, so that not everyone could just participate in the committee and you should not include a hundred people.” – (F, Interview 4)*

*“You know, because there are medical doctors, academics, scientists and patients represented [in the committee], but ordinary Dutch parents, who know nothing about it… It is about the blood of their children. They could ask some critical questions that trigger further consideration about the possible consequences or the after-effects. So I think it would add something to the current committee.” (F, FG 6)*

On the other hand, some FG-participants and interviewees were critical of parental involvement because of time issues, lack of knowledge on NDBS, or overrepresentation of people with negative opinions.

## Discussion

The current study aimed to provide insight in new parents’ perspectives on NDBS policy. New parents expressed needs for easily accessible information, adequate communication on the retention and (potential) use of NDBS, clearly described safeguards for privacy, a more active consent process, regulation for research conducted with NDBS research, and parental involvement in decisions on secondary use.

By using sequential mixed methods we were able to present quantitative data as well as a more in-depth understanding of themes. Nonetheless, this study has several limitations. The survey is not validated and framing of questions and topics in the survey and in the interviews sometimes differed due to the difference of the nature of the research approach. All participants were higher educated than average, and women were overrepresented. However, we did succeed to organize interviews and FG in four different regions in the Netherlands to gather different perspectives. With the survey we were able to reach a relatively large sample size, while we did not check if respondents took the survey twice, we consider that a low risk due to the length of the survey and the need to fill out personal details to enrol in the raffle for the voucher. Both interviews and FG achieved data saturation.

New parents are generally not very familiar with retention and secondary use of NDBS [21]. While information on retention and secondary use is available, it is not always perceived as transparent. Bombard et al. (2012) emphasized it is especially of great importance to improve transparency about retention and secondary use of NDBS towards the public [30]. To improve transparency Nordfalk and Ekstrøm (2019) performed a scoping study on the frequency and purpose of secondary use of NDBS in Denmark [31]. Rothwell et al. (2018) performed an international scoping review on the number, types, and designs of research using NDBS [32]. A similar study might contribute to improving transparency in the Netherlands^1^ and other countries or regions.

Other studies found distrust in authorities because participants believed that the government would store the NDBS longer than was communicated [22, 30, 33], but our study found a high trust in the RIVM to manage the NDBS and in academic organizations to conduct NDBS research. This high level of trust might contribute to the low percentage of Dutch parents who object to retention of their child’s NDBS for secondary use, which was 5.3% in 2017 [10]. Nonetheless, we found that participants did not consider current retention and secondary use of NDBS as fully anonymous, in line with perceived privacy risks reported by Botkin et al. [21]. These worries were especially expressed with respect to cybercrime against government systems. Despite the perceived risks, participants did perceive benefits of secondary uses of NDBS from a public health perspective, which is in line with previous studies pointing towards altruism [6, 21, 30, 34].

Engaging parents in decision-making has been discussed by several authors [18, 19, 35, 36]. Douglas et al. (2012) have also highlighted that parental and public involvement in research practices could improve research support in this area [11]. Our study participants have also expressed support for some form of parental involvement concerning secondary use. While approaches such as democratic dialogue techniques or adaptive governance seem successful [35, 37], difficulty remains to translate public deliberation into policy [18, 36]. Questions on translating outcomes of public engagement to policy remain undecided, for example regarding the form and level of involvement, and balance with other stakeholder involvement in general. While parental involvement is a challenge, our study is an example of making parents’ views available for NDBS policy.

## Conclusions

Our study contributes to parental involvement regarding NDBS policy. As such it contributes to including parental perspectives in the current policy discussion on longer retention. Our study shows that prolonged retention could be a feasible option in the Netherlands if several prerequisites are met.^2^ Based on our results and other studies, an important prerequisite is transparent information for parents. Therefore, implementation studies involving parents are needed to design optimal timing, content, and approach towards informing new parents and obtaining informed consent [38].

## Additional files

Additional file 1: Interview guide for the focus groups, consisting of an agenda for the focus groups and scenarios. (PDF 178 kb)

Additional file 2: Interview guide for the individual interviews, consisting of the topics for the individual interviews. (PDF 295 kb)

Additional file 3: Coding list of the codes used for the focus groups and individual interviews. (PDF 193 kb)

## Data Availability

The datasets used and/or analysed during the current study are available from the corresponding author on reasonable request.

## Abbreviations

FG: Focus groups
NBS: Neonatal bloodspot screening
NDBS: Neonatal dried bloodspots
RIVM: Dutch National Institute for Public Health and the Environment

## Footnotes

1 Since 2018 RIVM lists the studies that have been or are being performed using Dutch NDBS publicly accessible on their website.

2 In 2018 RIVM has designed and put in practice a policy that allows prolonged retention of a child’s NDBS up to 16 years on parents’ request. The policy has been initiated with the support of pediatricians and patient associations and is in line with results of this study.
