# Supplementary material for: Parental perspectives on retention and secondary use of neonatal dried bloodspots: a Dutch mixed methods study
Source: BMC Pediatr. 2019 Jul 9;19:230. doi: 10.1186/s12887-019-1590-8 (PMC6615086; doi:10.1186/s12887-019-1590-8)
Supplement: Supplementary file 1 — Interview guide for the focus groups, consisting of an agenda for the focus groups and scenarios. (PDF 178 kb) [file 12887_2019_1590_MOESM1_ESM.pdf]

Additional file 1

Parental perspectives on retention and secondary use of neonatal dried bloodspots: a Dutch mixed methods study

Marleen E. Jansen PhD, Lion J.M. van den Bosch MSc, Marjolein J. Hendriks MSc, Mariska M.J. Scheffer MSc, Marie-Louise Heijnen PhD, Conor M.W. Douglas PhD, Carla G. van El PhD

Script Focus Groups

Retention and secondary use of Neonatal Dried Bloodspots (NDBS)

Program

- 18.45-19.00 - Entry
- 19.00-19.10 - Introduction
- 19.10-19.20 - Background information
- 19.25-19.55 - Scenario 1
- 19.55-20.05 - Break
- 20.05-20.35 - Scenario 2
- 20.35-20.50 - Post-it round
- 20.50-21.00 - Closing

Roles

Facilitator: introduces the meeting, presents the scenarios, leads the discussion and the post-it round, closes the meeting.

Observer: set-up and check the equipment, observes participants during the discussion, manages the time, presents background information on the heel prick, collects the post-it's.

Aim

The aim of the focus group is to find out which policy on retention and secondary use of heel prick cards (NDBS) would receive the most support from citizens. Especially the argumentation is important. It is also important to find out if, how and when new parents would like to be informed about this matter.

Introduction (10 min)

- Welcome
- Introduction team members
- Explain the aim of this meeting

- Explain the rules with respect to confidentiality, audio recording (deleted after transcribing)
- Signing Informed consent
- Start audio recording (*observer*)
- Introduction participants including: how long are you pregnant/ how old are your kids and why are you here?
- Explaining the program:
    - Rules for discussion: confidential, respect everyone's opinions, don't interrupt etc.
    - Program: scenarios are hypothetical.
- Questions?

Background information (10 min.)

Short presentation about heel prick screening, retention and secondary use.

Scenario 1 (30 min.)

Hanna is pregnant with her second child. Time flies! Her due date is in 5 weeks, on May 15, 2022. She received information about the heel prick from the midwife. She had forgotten already that a heel prick will be done within the first days after birth, so she went looking for additional information online. She read that the heel prick tests for serious, congenital, rare diseases. She also read that anonymous blood, remaining from the heel prick, can be used by universities and companies for research into the **improvement of tests** used in the heel prick. Furthermore, she read that it can be used to investigate how often certain genetic characteristics occur that can cause diseases in childhood.

The midwife had said that since 2020 the heel prick cards will be stored for 16 years, but that Hannah can indicate if she does not want the card to be stored. If **companies** are allowed to use heel prick blood, Hanna **hesitates** whether or not she would give her consent. She starts a conversation with her friend Mariska about this. Because Mariska has objected to storing of the heel prick card of her son, it has been destroyed after 1 year. Mariska actually thinks it should be the other way around: people should **explicitly give permission** to store the card, otherwise it will be destroyed. Hanna decides to ask the midwife, during the next visitation, **what kind of research** is done with the cards and why they have to be kept for **16 years**. Hopefully the midwife will have the answer to this.

- Read (aloud)
- Request participants to write down their thoughts: mention 2/3 things that stand out (agree / disagree or raises questions).
- Emphasize that this is a possible future scenario
- Discuss

- If necessary, raise questions about:
    - Research by company vs. University?
    - What kind of research do the participants think of?
    - Public health: research for heel prick purposes or general?
    - Individual health: child or family
    - Victim identification
    - How do the participants view the opt-out method?
    - What information do they expect from their midwives and when?
    - Do they want to receive information earlier?
    - Use of brochures/ internet?
- Optional: Which term do they find desirable for storage / is 16 years a good term and why (not)?

Mention that from May/June the RIVM will publish approved research topics on the website.

Break (10 min.)

- Stop audio and ask participants not to talk about the matter during the break.
- Consultation between facilitator and observer.

Scenario 2 (30 min.)

Dion and Yasmine's neighbor recently gave birth. When Dion visits his neighbor to congratulate, he asks how the baby is doing. Neighbor Anneke says that things are going well! Last week he had the heel prick; he obviously did not like the sting. It reminds Dion of the moment his twin daughters had the heel prick, that indeed lead to two crying babies. Luckily, there was nothing wrong with them.

While picking up the twins from day care, he bumps into his friend Hans. Dion asks Hans how their son is doing. Hans tells that a lot of **research** is being done into the **rare condition** that Daan has been diagnosed with one year after birth. Researchers are now investigating whether in the future they could trace the disease early with the heel prick. Then they would be able to start treatment earlier with other children. Dion asks Hans if he is involved in the research since he knows so much about it. Hans explains that he is a **member of the association for parents** with a child with a rare disease. The association is represented in the committee that approves or rejects research proposals. Research proposals are submitted at the National Institute for Public Health and the Environment, which stores the heel prick cards.

In the evening, Dion discusses the matter with his girlfriend Yasmine. He is wondering what happens with the heel prick cards of his children; after all, those cards belong to their children. Yasmine is less concerned about this: the cards are **anonymous**, and she thinks that the government will handle it carefully. Dion would want to have a say on how to use heel prick cards, but does not want to be a member of some sort of advisory group. He will explore the possibilities of joining an online **panel** or **survey** in which research proposals will be discussed.

- Read (aloud)
- Request participants to write down their thoughts: mention 2/3 things that stand out (agree / disagree or raises questions).
- Discuss
- If necessary, raise questions about:
    - What kind of participation do the participants see for themselves and why? Parents of patients versus all parents? Commission or panel?
    - Who do the participants perceive to be the cards owner?
    - Why should the RIVM store the cards or should another organization store them?

Post-it round (15 min.)

Reason secondary use + who	***Storage period + who***	***Informed consent (opt-in/opt-out)***	***Involvement***

Copy the above table on a flip chart (observer). Use the powerpoint presentation to explain that everyone will stick one post-it per box that represents their final position/opinion on that aspect. First let the participants think for themselves. Show the examples on the powerpoint if necessary. Then briefly return to the answers and summarize.

- Do they agree with the reason for use?
- Who uses the cards?
- How long should they be stored?
- When should parents be informed/involved about this and how (opt-in /out)?

See powerpoint for possible options. The options currently being discussed are 4 or 16 years.

Closing (10 min)

- Thank you for the presence and input provided
- Travel costs reimbursement and completing demographic questionnaire
- Hand out the gift and brochure of the heel prick
- Questions or remarks?
