# Supplementary material for: Parental perspectives on retention and secondary use of neonatal dried bloodspots: a Dutch mixed methods study
Source: BMC Pediatr. 2019 Jul 9;19:230. doi: 10.1186/s12887-019-1590-8 (PMC6615086; doi:10.1186/s12887-019-1590-8)
Supplement: Supplementary file 2 — Interview guide for the individual interviews, consisting of the topics for the individual interviews. (PDF 295 kb) [file 12887_2019_1590_MOESM2_ESM.pdf]

Additional file 2

Parental perspectives on retention and secondary use of neonatal dried bloodspots: a Dutch mixed methods study

Marleen E. Jansen PhD, Lion J.M. van den Bosch MSc, Marjolein J. Hendriks MSc, Mariska M.J. Scheffer MSc, Marie-Louise Heijnen PhD, Conor M.W. Douglas PhD, Carla G. van El PhD

Interview guide individual interviews

INTRODUCTION AND PURPOSE OF THE RESEARCH

Good morning, this is ____ (introduce interviewer) from VUmc. I would like to thank you once again for your interest in the research on heel prick cards. For this research I am doing interviews to gain more insight into the opinion of the Dutch public on the storage and use of the heel prick cards for other purposes, such as further scientific research. I am particularly interested in your thoughts about the answers you have given in the previously completed survey. The insight that is gained on the public opinion can be used by National Institute for Public Health and the Environment (RIVM) for a new policy proposal concerning the use of heel pricking cards.

I will first briefly give some information about the heel prick. As you probably know, blood is collected during the screening on a heel prick card. These heel prick cards are stored for one year in a regional lab for quality control and then for another four years at a central location at the RIVM for use in research, such as research into the mapping of diseases. Thereafter, the heel prick cards are destroyed. Proposals for further research are assessed by a RIVM committee. The Center for Population Screening of the RIVM is responsible for managing the cards and making decisions for further research. The research that is currently permitted is for the improvement of the heel prick screening and / or the treatment of heel prick diseases. Heel prick material is only used when parents have not objected to the use. Objection to retention and secondary use is collected on the heel prick card through a signature. The material is only used anonymously. Do you have questions that you would like to ask before we start?

The interview will take about half an hour of your time. I would also like to emphasize that if you do not want to answer a question you are not obliged to answer and that all results of the interview are treated confidentially. If it’s okay with you, I will now start the sound recording.

TOPICS AND (MAIN) QUESTIONS
- Experience with heel prick screening
- Permission for secondary use
- Relationship secondary purposes and the length of storage heel prick card
- Management heel prick card
- Decision making about secondary use
- Information provision process

Topics	Questions
**Experience with heel prick screening**	*Main question:*<br>• Could you briefly describe what your experience is with the heel prick screening?<br>*Sub question:*<br>• Did you, at the collection of the blood spot, object to the use of the heel prick card (s) for scientific research?
**Permission for secondary use**	*Main question:*<br>The heel pricking cards may currently only be used for further research that improves heel prick screening and for the treatment of heel prick diseases when there is no alternative. This research is approved by a committee of the RIVM.<br>• What do you think of the use of the remaining heel stimulation cards for these purposes? (Ethics / Trust)<br><br>*Sub questions:*<br>In the future, the cards could also be used for other purposes.<br>• If you are allowed to decide for which purposes the heel prick cards may be used, for what purposes would that be? (Ethics)<br>• In your opinion, what are the prerequisites for using the heel prick cards for further research? (give explicit permission / anonymous) (Ethics)<br>• Could you describe a situation when, according to you, the heel prick cards should not be used? (Ethics)<br>• What positive / negative aspects do you think exist when the heel prick blood is used for further research? (Ethics / Perceived Risk)<br>• To what extent do you find anonymous use of the heel prick cards important in the case of scientific research? (Ethics / Perceived Risk)<br>• Who should be allowed to use the heel prick cards? (government / companies / universities). (Trust / Knowledge)
**Management heel prick card**	*Main question:*<br>Currently, the maximum storage of the heel pricking cards is five years and is stored at a central location at the RIVM for further investigation.<br>• Could you explain why you are or are not satisfied with the current management of the heel prick cards? (Trust / Knowledge)<br>*Sub question:*<br>• If you could decide on the length of the storage, what would this look like? (Ethics)

**Relationship secondary purposes and the length of storage heel prick card**	*Main question:*<br>• If the length of storage were to change, do you think the length of storage should be dependent on the purpose potential studies? (Ethics)<br>*Sub question:*<br>• Can you elaborate on this using an example?
**Decision making about secondary use**	*Main questions:*<br>As mentioned before, a committee of the RIVM assesses research proposals concerning the heel prick cards. This committee consists of a multidisciplinary group of people involved with the heel prick screening or with expertise in the field.<br>• What do you think of the participation of the public in this committee in the decisions regarding the use of the cards for further research? (Personal control)<br>• To what extent would you want to influence the decision-making yourself? (commission / online panel / online questionnaire) (Personal check)
**Information provision process**	*Main question:*<br>• Could you explain at what times you would like to be informed about the use of the heel prick cards for other purposes? (Ethics / Knowledge)<br>*Sub questions:*<br>• Would you like to be informed when the card is used for scientific research? Can you explain a number of reasons why you think this is important? (Ethics / Knowledge)<br>• In addition to being informed of the use, would you also like to be informed about the results of the research? To what extent do you consider this important? How would you like to be informed about this? (Ethics / Knowledge)<br>• In order to inform parents more about the storage and use of the heel prick card, the personal data must be stored separately. To what extent do you consider the provision of information to be more important than maintaining anonymity? (Ethics / Knowledge)<br>• How do you think about a no-unless (opt-in) system, so when you have to give explicit permission?

CONCLUDING REMARKS

Is there anything else you would like to share with regard to the subject? Any further questions that you would like to ask?

I would like to thank you very much for sharing your opinion and participating in the interview. Within a few days I will send a summary of the interview to you so that you can check the accuracy of the information.
