# Supplementary material for: Parental perspectives on retention and secondary use of neonatal dried bloodspots: a Dutch mixed methods study
Source: BMC Pediatr. 2019 Jul 9;19:230. doi: 10.1186/s12887-019-1590-8 (PMC6615086; doi:10.1186/s12887-019-1590-8)
Supplement: Supplementary file 3 — Coding list of the codes used for the focus groups and individual interviews. (PDF 193 kb) [file 12887_2019_1590_MOESM3_ESM.pdf]

Additional file 3

Parental perspectives on retention and secondary use of neonatal dried bloodspots: a Dutch mixed methods study

Marleen E. Jansen PhD, Lion J.M. van den Bosch MSc, Marjolein J. Hendriks MSc, Mariska M.J. Scheffer MSc, Marie-Louise Heijnen PhD, Conor M.W. Douglas PhD, Carla G. van El PhD

Interview Data Coding Scheme

1. **Ethics**
   - 1.1. Transparency
     - 1.1.1. Sharing information about the use of RDBS
     - 1.1.2. Sharing information about the storage of RDBS
   - 1.2. Altruism
   - 1.3. Autonomy
   - 1.4. Privacy
   - 1.5. Anonymity
     - 1.5.1. Use of personal information during research RDBS
     - 1.5.2. Use of personal information results secondary use RDBS
2. **Knowledge**
   - 2.1. Knowledge on newborn screening
   - 2.2. Knowledge about secondary use of RDBS
   - 2.3. Knowledge on retention period RDBS
   - 2.4. Knowledge on management RDBS
3. **Trust**
   - 3.1. Trust in use RDBS
     - 3.1.1. Trust in the authority storing RDBS
     - 3.1.2. Trust in universities using RDBS
     - 3.1.3. Trust in companies using RDBS
   - 3.2. Trust in storage RDBS
   - 3.3. Trust in decision making use RDBS
4. **Perceived risk**
   - 4.1. Negative aspects secondary use RDBS
   - 4.2. Negative aspects prolonged retention
   - 4.3. Negative aspects management RDBS

5. **Perceived benefit**
    5.1. Positive aspects secondary use RDBS
        5.1.1. Promotion individual health
        5.1.2. Promotion public health
        5.1.3. Increase of knowledge
    5.2. Positive aspects prolonged retention
        5.2.1. Promotion individual health
        5.2.2. Promotion public health
        5.2.3. No sample ‘squandering’
    5.3. Positive aspects management RDBS
6. **Personal control**
    6.1. Want to give permission secondary use RDBS
    6.2. Personal control in decision making process secondary use
    6.3. Public control in decision making process secondary use
    6.4. Receiving information
        6.4.1. Wanting to receive information about the results of initial screening
        6.4.2. Wanting to receive information about the moment of use RDBS
        6.4.3. Wanting to receive information about the results RDBS use
7. **Attitude**
    7.1. Attitude towards newborn screening
    7.2. Attitude towards consent procedure
    7.3. Attitude towards RDBS of newborn screening
        7.3.1. Attitude towards the retention period RDBS
        7.3.2. Attitude towards secondary RDBS use in general
        7.3.3. Attitude towards extended research uses RDBS
        7.3.4. Attitude towards management RDBS
        7.3.5. Attitude towards decision making
8. **Others**
    8.1. Experience with newborn screening
    8.2. Involvement

(total: 56 codes)
